# Observations on the Pathology of Venereal Affections

**Published:** 1830-07-01

**Authors:** 


### OBSERVATIONS ON THE PATHOLOGY OF VENEREAL AFFECTIONS. By BENJAMIN TRAVERS, F.R.S. &c.*

This little work on the venereal is merely the offspring of an anniversary meeting of the members of the Hunterian Society, arrayed in a suitable tegument of pasteboard, and affiliated to those proprietors of so many hot-pressed bantlings, Messrs. Longman and Co. It is not to be expected, under these circumstances, that the foundling, should have swelled into the goodly corpulency of a full-sized octavo; we might as well expect that the shy miss should display, on her first coming out, the mature coquetry and confident ogle of the trained and *accomplished* madam. But to drop the metaphor, Mr. Travers' brochure is a short but shrewd essay, containing many points which are worthy of attention, and in some instances open to dispute. It is not consistent with the objects of this Journal to enter on the analysis of works on syphilis, but we cannot refrain from noticing some parts of the present essay, that we hope will not prove unacceptable to our readers.

### GONORRHŒA.

Mr. Travers observes that a purulent discharge from the male urethra or female vagina, occurs not unfrequently independent of sexual intercourse, and, as a sympathetic affection, may exist even in infancy. This should be borne in mind by practitioners, for several instances of serious consequences, from ignorance of this fact, have been recorded. The purulent discharge from the vagina of young children has been looked on as evidence of violation: indeed, if we mistake not, such a blunder was made very lately by a medical man, and gave rise to unpleasant discussions in the newspapers. The vaginal mucus of a maiden woman may be converted, by any inflammatory action of the parts, into puriform fluid; and attempts to deflower female children have been frequently found to produce a purulent secretion from the vagina, independent of any gonorrhœal taint in the offending party.

"I believe that the vaginal secretion in either of these, or similar cases, is capable of communicating the inflammatory irritation to other mucous surfaces, either of the same or another individual. I have seen many cases of acute suppurative inflammation in the eyes of new-born children, where I was well convinced that the mother could not be the subject of gonorrhœa, and others, in which the existence of that disease was indirectly ascertained, though it could scarcely have been suspected; but I never met with a case in which, upon strict inquiry, neither this nor the inflammatory leucorrhœa, so often attending upon advanced pregnancy, was not ascertained to be present. It is well known, that a woman so affected sometimes communicates a discharge to her husband; the case being not so rare, in reputable classes of society, as to render the fact doubtful to experienced surgeons."

From these premises, then, our author concludes, that the affection termed gonorrhœa is not *necessarily* to be referred to any specific quality of the matter. This is certainly true, but we believe that it *ordinarily* depends on a specific contagion. Mr. Travers, however, brings forward arguments to prove that gonorrhœa is of a simple inflammatory nature, and that "its ordinary origin is from the irritation of purulent matter. I say 'ordinary,' because we have seen that the disease may arise independent of intercourse, or after connexion with a sound female, the

---

* Octavo, pp. 75. London, 1830.

urethral membrane being already irritated, or even inflamed, but not discharging purulent matter." If we perfectly understand the foregoing proposition, we have our doubts of its absolute soundness, nor do we think that the arguments on which it is bottomed are unanswerable. But n'importe; we pass to another point.

"Hitherto I have spoken of the gonorrhœa, strictly so called, viz. the inflammatory secretion from the male urethra and female vagina. So long as sound surfaces remain on both sides, it is my belief that no secondary symptom of a specific character follows; that in fact no poison is formed. I do not say, that no such quality belongs to the matter which may be secreted by unbroken surfaces. Much of the difficulty of this subject has arisen from the supposition that either gonorrhœa or sores, or both, are the product of a particular virus or poisoned matter, and that a gonorrhœa necessarily gives a gonorrhœa, and a sore, a sore. This is altogether erroneous. Inflammation is excited by the irritation of matter from the inflamed follicles of the sound surface as well as from the ulcerated surface, and the difference of its effects upon the party who receives it, depends exclusively upon absorption or non-absorption, *i.e.* the formation or absence of a sore, a circumstance often accidental."

The proper gonorrhœa, or secretion from the unbroken mucous membrane of the urethra is incapable, says Mr. Travers, of producing secondary symptoms; but if an excoriation or an ulcer (gonorrhœal) be present, the matter which it secretes is capable of producing, by its absorption, secondary symptoms in the individual. "The absence of secondary symptoms in pure gonorrhœa depends, therefore, not upon any difference in the quality of the matter, but upon a law of the animal economy, *that the inflammatory secretions of the sound surface are not absorbed into the system.*" Now we rather think that this doctrine is heterodox, that it is a heresy, and a keen disputant might shew, without much difficulty, that it leads to the notion of a similarity, if not consanguinity, between the gonorrhœa and the lues. This at least is certain, that unless the characters of the gonorrhœal sore be distinct and distinguishable, without much uncertainty, from the syphilitic chancre, they must come in practice to be treated as the same; and this will apply with even more force to the secondary symptoms, for unless the diagnosis of the syphilitic and the gonorrhœal be tolerably easy in in itself, we shall merely have the *history* of the primary sore for our guide, a venereal history fallacious to a proverb! The following, then, are the distinguishing marks of the gonorrhœal sore and secondary symptoms, which we give in our author's own words.

"The distinguishing features of sores produced by gonorrhœal matter, are circularity, flatness without induration, whether raised or level with the surface; seldom solitary, often several; their greater frequency on the anterior and posterior verge of the prepuce, or beside the frænum; *i. e.* at the angles of reflection between the layers of the prepuce, or the close and loose investment of the glans, than elsewhere. In the female, they are likewise commonly situated at the junction of the mucous with the cuticular membrane upon the labia, or at their inferior commissure. Their margin is blunt but not indurated, the character of the granulatiou is spongy and indolent, and though they clean readily, they heal slowly. Lunar caustic, lightly applied, quickens them; and the solutions of caustic, copper, zinc, and alum, escharotic astringents, are among the best local remedies.

"The secondary symptoms of the gonorrhœal sore are as strongly marked, and present as distinct a character as those of lues. The glands in the groin are oftener enlarged and indurated, than otherwise, in protracted cases; but, as in proper gonorrhœa, the affection is sympathetic. The appearance of secondary symptoms is certainly not peculiar to these cases. The inflammation of the velum palati and uvula is diffuse and superficial; the surface is roughened with innumerable small tufts of white lymph, or pitted with small and shallow indentations, where ulceration has taken place. These are so slight as often to escape ordinary observation. They are seen chiefly upon the tonsils, uvula, apex and edges of the tongue. The sharp, deep and clean fissures of the tonsil, like the roughened and pitted tonsil, are consequent upon gonorrhœal ulcers of the genitals; but this appearance is later, and I am disposed to think, induced by the partial and alterative action of mercury upon the system; in other words, a progressive stage towards the cure. The gonorrhœal sore throat is accompanied by considerable irritability, to stimulant fluids especially. The excavated ulcer of lues, with its abrupt high-coloured margin, is not more strongly characterized, or more readily distinguished. The cutaneous affections are slight, and in character presenting less variety than those of lues, so far as my observation enables me to speak. The papular and squamous are the most common, the pustular and tubercular, occasional. The lichen and psoriasis upon the trunk and limbs, and the achor and acne indurata, thickly distributed upon the face and verge of the hairy scalp, are the forms which I have chiefly recognized. The attempt to discriminate and class the minute varieties of primary sores, and to establish corresponding determinate forms of cutaneous eruption, appears to me to suppose an uniformity at variance with observation, if not with nature, and to render what is sufficiently clear for all practical purposes, studiously obscure. General character is a sure and sufficient guide. This will pretty infallibly distinguish the gonorrhœal sore from the chancre, the gonorrhœal sore throat from that of chancre, and in most instances, the eruptions consequent upon either, from the eruptions produced by mercury. I have not, however, attained such nicety of discrimination, as to pretend to determine the character of the primary sore from that of the eruption, with any feeling of confidence. The scrotum is frequently the seat of scabbing excoriations and eruptions of a peculiar character, connected with a morbid thickening and elongation of its extensile cutis. These, I believe, are generally depending upon direct irritation. The verge of the anus likewise presents peculiarly characterized eruptions, which I have been sometimes disposed to regard as resulting from a similar cause."

So much for the sore and the secondary symptoms of gonorrhœa, and our author next adverts in succession to the inflammation of the conjunctival palpebra, tenderness and soreness of the flat bones, affections of the joints, &c. and finally that destructive malady gonorrhœal ophthalmia. Mr. Travers remarks that the gonorrhœal poison, like the small-pox, is communicated to the fœtus in utero by an infected mother, and likewise to the infant at the breast. Ulcerations of the mucous membrane of the nares, eyes, mouth, pudenda and verge of the anus, with papular eruptions and blotches upon the skin of the infant are the more ordinary symptoms. They yield to the hydrarg. c. cret. three or four grains night and morning. The succeeding directions with regard to treatment conclude the subject of gonorrhœa.

"Purgatives, sudorifics, diuretics and mucilaginous diluents, rest, and frequent tepid ablutions more than half cure the disease by removing the cause. Over the running no medicine has any power to be compared with the copaiba. Of injections, when the discharge is gleety, none are equal in my experience to those in common use, of lead, copper, and zinc. The inflammation of the cervix vesicæ, spasmodic stricture, swelled testicle, phymosis, with irritable sores at the corona glandis, thickened and ulcerated prepuce, burrowing abscesses between the bodies and integument of the penis, and warts, in nine cases out of ten, are consequences of inattention to cleanliness, or exasperation from a totally wrong treatment in the early stage of the complaint.

"Neither the sores nor the secondary effects of the gonorrhœal poison require more than the alterative tonic action of mercury; by its full action they are impeded, as it irritates and depresses the system disadvantageously. I will not say that the constitutional symptoms may not be cured without mercury; but the result of my experience is that the gentle action of that medicine, such as is given by Plummer's pill, the oxymurias hydrarg., and in some feeble systems by the hydrarg. cum cretâ, so materially expedites the curative process, that independently of any specific efficacy, real or supposed, I avail myself of its assistance. I am guided by its influence on the disease as to the extent and continuance of its use. The disadvantages of slowness in the cure and a continual tendency of the disease to relapse, or reappear in a new form, long since compelled me to abandon as a general principle, that of treating these cases without mercury. The sarsaparilla I almost invariably give at the same time in substance and decoction simple or compound; and in cases so slight, that I think the alterative not called for, or perhaps already sufficiently administered, I give with it free doses of the diluted nitric acid, with or without an equal portion of the tincture of henbane.

"If much irritability is present, especially in the throat affections, the sublimate, combined with small doses of the extract of conium or opium is an estimable form, and where constitutional debility prevails, the hydrarg. cum cretâ with a little rhubarb or Dover's powder, as the case may require. The indication of treatment is twofold, alterative and tonic; if much pain and irritability be present, a sedative should be added. The readiness with which the disease yields to this plan, steadily supported, it is most gratifying to witness."

### LUES OR SYPHILIS.

Mr. Travers attempts with considerable ingenuity to prove, that the gonorrhœal and venereal poisons differ only "in the degree of their intensity and the extent of their operation." We have said that his arguments are ingenious, but to us they are far from conclusive, indeed had we a mind for polemical discussion we think it would not be impossible to prove them fallacious. But since arguments on these subjects, when sown upon paper, breed disputes as countless as the warriors that sprung from the dragon's teeth, we shall decline them altogether. We do so on the present occasion with the less reluctance, as a revival of the Hunterian affinity between gonorrhœa and syphilis is not likely to be re-admitted into favour without keen examination.

For thirty or more cases of gonorrhœal sore, we now have but one of eating chancre, and the question arises —to what ought this to be ascribed? Let Mr. Travers answer it.

"Judging from the records of history, there is much reason to believe that in the course of centuries the disease has been essentially altered in character, and certainly it has been subjected to no influence so direct and powerful as that of mercury. It is, perhaps, as much because that remedy has formerly been so indiscriminately and freely employed, as of late years so sparingly exhibited, that we find the disease comparatively so little formidable. There is, however, ground to believe, that what we may have gained upon the disease by mercury, has been obtained at the expense of life and limb. The poison has been bereft of its virulence at the cost of the constitution, and for the cure of syphilis in an individual, scrofula has been entailed upon his posterity."

Another important fact is the daily amalgamation between the syphilitic and gonorrhœal sores, an amalgamation which is throwing down the barriers of distinction between them, if indeed they are not already shattered, and which renders the administration of a certain proportion of mercury "unquestionably the safer practice in all sores." This admission on the part of Mr. Travers is a significant illustration of our remarks, and an ample apology for not having taken up the gauntlet.

"The great distinction of the syphilitic ulcer, primary and secondary, from the gonorrhœal, is that the inflammation of the former is deep instead of being superficial and erythematous, and the colour so intense as to approach lividity. The ulceration is exceeding or depascent, instead of herpetic, extending equally and rapidly both in depth and circumference. Formerly I have seen two-thirds of the glans penis disappear in less than a week in acute cases; but these are now rare. Central sloughs are formed, but it is not by sloughing that it extends; the edges are sharp and abrupt, not shelving. The syphilitic ulcer has none of that shifting character which belongs to lupous, carcinomatous, and cachectic sores, viz. cleansing and cicatrizing in one part and spreading in another. Its extension is in a direction from the surface. It is only when exasperated by local irritants or stimulating regimen, and that in a debilitated habit, that the syphilitic ulcer turns sloughy; its character is purely ulcerative, not gangrenous phagedæna."

Mercury is the specific, but two states of constitution absolutely prohibit its immediate use;—1st. excessive inflammation; 2d. excessive weakness. In the first the usual antiphlogistic measures are required, and in the second the extract. sars. dissolved in the decoction, or if a higher tonic be required, the same with bark, and a free allowance of nutrient food, wine, or porter.

"If ulceration is making rapid strides the better plan is to introduce the remedy by the skin in frictions, night and morning; and if the system resists its entrance, to aid the process by the pill. In cases of great debility, I begin with the oxymuriate or the mercury and chalk, as a test of the capability of the system to bear it. The anodyne, if need be, and the tonic of course should be continued. In most cases mercury and bark or sarsaparilla are exhibited with excellent effect at the same time. In ulcers of the throat, fumigations are of the greatest efficacy. I often depend upon them alone in weakly persons, while other medicines are directed to the support of the system. They effect an improvement more rapid in these cases than the constitutional action alone. I should say generally, that to render the action of mercury powerful over the disease, and to preserve the system from its injurious operation, the support of the patient's strength becomes the principal object of the surgeon's attention. Indeed, the successful treatment of the disease turns chiefly upon his knowledge and consistent pursuance of this indication."

The treatment of venereal ulcers requires the same attention to prevailing character as other sores; excessive irritability is best allayed by a saturated watery solution of extract of opium. In sloughy ulcers of the throat the linimentum æruginis is most effectual. We need not advert to the secondary symptoms, but may conclude this part of the subject, by stating in our author's words, that the profuse and wasting action of mercury is *never* called for.

Compound of Syphilis and Mercury.

Such a combination, especially when aggravated by the presence of scrofula original or engendered, gives rise to cachexiæ of the most formidable kind. Cold and dram drinking are the ordinary exciting causes, the abuse of mercury and the dregs of syphilis the predisposing. Excepting the eczema, a species of ecthyma, and the impetigo rodens, Mr. Travers has no acquaintance with eruptions proper to mercury as a single agent. That mercury has been frequently used to an injurious extent, he admits without hesitation, but still he objects to the terms 'mercurial eruption,' 'mercurial sore-throat,' 'mercurial pains,' as equivocal in meaning, and therefore improperly used.

"From this source are doubtless derived the caricature portraits of the venereal disease abounding in the records of medicine and the reports of empirics; and I may add the cases, not now so numerous as formerly, of direful and sometimes fatal deformity in the foul wards of our hospitals. The emaciation, pallor, fetor; the deep, eroding, foul ulcers; the worm-eaten bones, as of the whole cranium; the rupia in the form of conoidal limpet-shell crusts covering the body; the continuous slough of the whole posterior fauces, extending beyond sight; the entire loss of the parts of generation; of the soft and hard palate, and the falling in of the nose; the agonizing night pains, the severe hectic fever and excessive and offensive sweats, &c. sufficiently characterize these cases. Their termination is ordinarily in phthisis or hæmoptysis, or some special visceral disorganization. No remedy, next to the adjustment of a diet as generous as the patient can take, is equal to the extract of sarsaparilla in these cases. The infusion of the root in lime-water is a form admirably adapted to a weakened stomach, and with this fresh milk may be advantageously combined in equal proportions; but the extract dissolved either in its decoction, in milk, or in lime-water, to the amount of half an ounce per diem, or more, is the restorative upon which I rely in these cases. Its power is most extraordinary, more so than that of any other drug with which I am acquainted. *To regard it as inert, as a mere diluent or an offensive nutrient, is either a proof of a very limited experience or a very prejudiced observation.* It is in the strictest sense a tonic, with this invaluable attribute, that it is applicable to a state of the system so sunken and yet so irritable, as renders other substances of the tonic class unavailable or injurious."

We have taken the liberty of marking in italics the passage respecting sarsaparilla, because we most cordially concur with Mr. Travers in the sentiment which it contains. To consider sarsaparilla as an inutile lignum, a thing no better than saw-dust, appears to us to be the acmé of prejudice, the wild fanatic scepticism of a book-learned theoric. But our space is nearly consumed, and yet we are unwilling to close this short review without enriching our pages with a graphic description of a frightful form of venereal affection; it is the 'Swan-alley sore.'

"I shall avail myself of this opportunity to notice a peculiar and very formidable distemper, arising from the unlimited intercourse of young and delicate girls of scrofulous temperament, chiefly with foreign sailors, many of them lascars or men of colour, frequenting the brothels in the vicinity of the East and West India and London Docks. The district of St. Catherine's (until recently converted into docks) was the most notorious for the propagation of this pestilence, and a place in that quarter called 'Swan Alley,' has given the sore that appellation in St. Thomas's Hospital. The subjects of the disease are almost exclusively females. I remember only one instance of a boy similarly affected, in whom the disease went unchecked to a fatal termination. The girls are slender, with very thin fair skins, and often light hair, and generally from 15 to 25 years of age. They have been a few months before decoyed by the Jews who keep these houses, and are systematically on the look out in the great neighbouring thoroughfares. The girls newly arrived in London, while in search of lodgings until they procure places, become victims to these miscreants.

"They receive the visits of as many men as there are hours in the day, and are supported on scanty food and abundance of gin. Their visitors do not always restrict themselves to natural connection. When they become constitutionally ill, their keepers send them to the hospitals. The Magdalen ward of St. Thomas's is seldom without one or more of them. They have been only two or three days in the house, when the character of the sore displays itself; for by reason of the previous illness they are rarely detained in their occupation long enough for the ulcer to have assumed its genuine features. It is a circumscribed irregular ulcer with an inflamed blunt edge, usually situated at the lower angle of one labium, or in the cleft of the nates. When the sore inflames, its edge acquires a dark crimson colóur to some distance around; the surface is covered with a deep, tenacious, ash-coloured slough, and it extends so rapidly, as to be increased visibly from day to day. It is generally attended with excessive unremitting pain, a very rapid and contracted pulse, great paleness of the surface, total failure of the appetite, and great depression of strength and spirits. It is, in fact, acute gangrenous inflammation. Where they recover, no secondary symptom of lues appears; nor is the disease in any degree contagious. The treatment now adopted seldom fails to arrest it, unless admitted in a very advanced stage, as after the sloughing process has been some time established when the devastation is truly terrific. In addition to the slough of the pudendum, I have seen the entire lower opening of the pelvis deprived of its soft parts. The girl dies typhoid with a dry black tongue, and is first delirious, then comatose.

"When the pain is severe and the disc of inflammation strongly marked, blood-letting is beneficial to both. I usually apply lint soddened in a saturated solution of the extr. opii, over this a poultice of linseed meal, and cover the whole with a fomentation flannel. This seldom fails to relieve, if not to remove the pain. The exposure of the sores and the change of dressings much augment it; the continued application of warmth and moisture as much abate it. After clearing the bowels with castor oil, I gave a draught of camphor julep with a drachm of ether, and ten minims of the tinct. opii every four hours; and half a grain of opium additionally, if the pain is very urgent. If the slough is fast and the ulcer extends, the surface is washed freely with the strong nitric acid, and it is remarkable that very shortly afterwards the girl expresses great relief. The London treacle poultice I likewise find an excellent application, covered by the fomentation flannel. The object to be looked to for directing the application, is the colour of the surrounding skin; when this pales, the dilute nitric acid lotion, ten drops to an ounce of water, is the best application. Fresh eggs and milk, and as the stomach acquires tone, a mutton chop, and from ten to twelve ounces of port wine daily, are an appropriate support. The occasional repetition of the oil or the common enema should not be neglected under the habitual employment of opium.

"The strong acid must be repeated each third or fourth day, till the whole surface granulates. When the girl sleeps and takes nourishment, notwithstanding an immoderately quick pulse, she does well; and the sore, when once clean, heals rapidly under the dilute acid lotion and simple cerate. The bark is useful at this period, but very secondary to the opium, wine, and nutriment. The former should be gradually reduced. A lotion of the chloride of lime and caustic soda, three drachms of the first and one drachm of the last to half a pint of water, acts with magical celerity in clearing the sloughs in many cases; but I have not found it so applicable or efficacious during the stage of acute inflammation, as when it is subdued. I once saw mercury rubbed in to rapid salivation, with manifest acceleration of the destroying process, and the vital powers were further greatly sunk by it. I have seen the inflammation begin after the taking of half a dozen blue pills, one every night and morning, which had been prescribed upon the girl's admission for a sore, which was then small and indolent, in ignorance of its character and tendency."

Mr. Travers believes that this sore is neither the result of the irritation of the matter of gonorrhœa, nor merely a local disease. Bad constitutions, incessant local stimulus, want of clean linen, and the substitution of burning spirits for proper nutriment, induce as soon as ulceration appears this gangrenous phagedæna. The last point mooted by our able author is the origin of the venereal. Now we should just as soon dream of sailing to Cat Island, and invoking the manes of the Aborigines who first saw with wonder the Spanish caravals emerging from the bosom of the Western Ocean, as of ripping up this long-disputed question. It is very well for gentlemen with some leisure and much learning, but for us—morbleu! it would be perdition. We cannot conclude without expressing the gratification we have derived from perusing this little Essay of Mr. Travers', of which we have now extracted the marrow for our readers. We beg Mr. T. to accept our best acknowledgments.

